# Psychometric properties of the genderism and transphobia scale in Iranian students

**DOI:** 10.34172/hpp.025.43507

**Published:** 2025-05-06

**Authors:** Ali Reza Shafiee-Kandjani, Sara Tajlil, Asal Raeisnia, Şenol Turan, Behzad Shalchi

**Affiliations:** ^1^Research Center of Psychiatry and Behavioral Sciences, Tabriz University of Medical Sciences, Tabriz, Iran; ^2^Cerrahpaşa Faculty of Medicine, Department of Psychiatry, Istanbul University-Cerrahpaşa, Istanbul, Türkiye; ^3^Working Group of Psychiatry and Psychology Culture-Based Knowledge Development, Tabriz University of Medical Sciences, Tabriz, Iran

**Keywords:** Gender dysphoria, Gender identity, Reproducibility of results, Transgender persons, Validity, Reliability

## Abstract

**Background:**

Although the prevalence of transgender individuals has increased, there is currently no scale that aligns with Iranian culture to assess transphobia. Therefore, this study aimed to evaluate the validity and reliability of the Persian version of the Genderism and Transphobia Scale (GTS), and its cross-cultural adaptation in an Iranian setting.

**Methods:**

This psychometric study involved 418 students in Tabriz, northwestern Iran. Forward-backward translation was conducted to develop a Persian version of the scale. Reliability was assessed using test-retest, Cronbach’s alpha, and the interclass correlation coefficient (ICC) test. Face, content, and construct validity were also evaluated.

**Results:**

The Cronbach’s alpha was 0.91 for transphobia/genderism (T/G), 0.83 for gender-bashing (GB) and 0.91 for the total GTS. The results of the exploratory factor analysis (EFA) showed that the two factors, T/G and GB, collectively explained 47.05% of the total variance. The ICCs for different factors of the Persian-GTS including T/G, GB, and GTS total were r=0.79 (Confidence interval [CI] 95%; 0.86 - 0.90), r=0.65 (CI 95%; 0.71 - 0.80), and r=0.98 (CI 95%; 0.68 - 0.79), respectively. An EFA identified two factors defining 47.05% of all the variance. Items number 8 and 31 were removed from the Persian version of GTS.

**Conclusion:**

The Persian-GTS was identified to be valid and reliable for evaluating students’ attitudes toward transgender individuals. Consequently, the Persian-GTS can be utilized in research concerning health issues related to transgender individuals.

## Introduction

 Personal identity is determined by a variety of factors such as ethnicity, nationality, religion, occupation, and gender identity. Gender identity is individuals’ deeply-held sense of their own gender, which may or may not align with their biologically determined sex.^1^ Gender dysphoria (GD) is a new diagnosis in DSM-5 replacing the diagnosis of gender identity disorder (GID) in DSM-IV. According to DSM-5, GD reflects the distress and anxiety related to the incongruence between an individual’s gender identity and his/her assigned sex, lasting for a minimum of six months. This transition from GID to GD has played a role in reducing the relative stigma associated with transgenderism.^2^

 The prevalence of GD is approximately 1 to 3 per 100 000 individuals, with a male-to-female ratio ranging from 3-5 to 1. In Iran, the estimated prevalence of transgender women is 1 in 145 000, and for transgender men, the estimation is 1 in 136 000.^3^ It is estimated that about 4000 individuals living with GD in Iran.^4,5^

 In the United States, transgender individuals constitute approximately 0.5% to 6.0% of the total population.^6^ These individuals often encounter significant discrimination, violence, and harassment.^7^ Transgender people experience considerable social and interpersonal stigma, which can result in adverse mental and physical health outcomes.^7,8^ Additionally, they are often neglected within the healthcare system, and encounter multiple barriers, including limited access to services, social exclusion, discrimination, financial difficulties, socio-economic challenges, and a lack of awareness among healthcare providers about their circumstances.^9-13^ These individuals often receive inadequate support from healthcare professionals during their illness, highlighting the urgent need for greater attention to transgender issues.^14,15^ Numerous studies have documented discrimination and negative attitudes towards transgender people.^16-18^

 Given that healthcare for all is regarded as a fundamental health principle, and considering the rising prevalence of transgender individuals, it appears essential to understand society’s attitude towards these individuals. The Genderism and Transphobia Scale (GTS) is accepted as a reliable and valid questionnaire in English for assessing transphobia. To the best of the authors’ knowledge, no research on the psychometric properties of the GTS in Persian has been found. Therefore, this study aimed to translate and conduct the psychometric properties of the GTS among the Iranian population.

## Methods

###  Study design and setting

 The current psychometric study was carried out to evaluate the validity and reliability of the Persian version of GTS among Iranian students in 2023. Participants were the students of Tabriz University of Medical Sciences. A convenience sampling method was used to recruit participants.The inclusion criteria for the study comprised willingness to participate in the study, and being at least 20 years old.

###  Original version of GTS

 The original version of GTS was developed and validated in Canada in 2005 by Hill and Willoughby^16^ to assess genderism and transphobia. This scale assesses cognitive, emotional, and behavioral aspects of people’s attitudes towards transgender individuals. It constitutes 32 items divided into 2 components: 25 items measure transphobia and genderism and 7 items evaluate sexism. In the original version of the GTS scale, Cronbach’s alpha coefficient of 0.83 was reported by its developers for the sexism subscale. This coefficient was 0.94 for the transphobia subscale, and 0.79 for the gender panic. The developers have reported a Cronbach’s alpha coefficient of 0.95 for the whole scale. GTS is responded by a 7-point Likert-type scale. A lower score in this scale indicates less negative attitudes toward transgender people.

###  Phase one: translation of the GTS

 Initially, written permission to translate the GTS into Persian was obtained by sending an email to the original GTS developer (Professor Darry B. Hill). The translation process encompassed, according to the five-step translation guideline recommended by the World Health Organization: (1) forward- translation; (2) expert panel; (3) back-translation; (4) pre-testing; and (5) final version.^19^

 First, two bilingual translators independently translated the tool into Persian. To address discrepancies, the translated versions were compared and merged in the next phase. In the case of significant differences, a third translator made the final decision. In the third step, two native English translators retranslated the Persian version back into English. Then, the items required adjustments in terms of cultural and common health issues in the region were modified using expert opinions and literature reviews.

###  Scale score validity

####  Content validity

 Content validity was assessed using both quantitative and qualitative methods. For this purpose, an expert panel was convened, consisting of 13 experts (psychiatrists (n = 6), psychologists (n = 4), and health education specialists (n = 2)). Quantitative content validity was assessed by calculating the content validity index (CVI) and content validity ratio (CVR) of the items. Therefore, the experts completed the forms on the necessity of the items using the 3-point Likert scale (Not necessary, necessary but needs revisions, Necessary). The CVR was calculated for each item using the formula CVR = [ne − (N/2)] / (N/2), where “ne” and “N” represent the numbers of experts who selected the essential point for the item and the total number of experts, respectively. Regarding the number of experts who participated in the study and the Lawshe CVR table, the minimum acceptable CVR is 0.54.^20,21^ CVI indicates the simplicity, clarity, and relevancy of the items, and it consists of two types: Item CVI (I_CVI) and Scale CVI (S_CVI). I_CVI is related to each item and calculated using a four-point Likert scale (one = not relevant to four = highly relevant).^21,22^ For this purpose, the experts completed the forms that were specifically designed for CVI. In addition, I_CVI was estimated by dividing the number of experts who rated this item as three or four by the total number of experts. The S_CVI was computed by summing the I_CVI values and dividing them by the number of items. A minimum of 0.79 and 0.80 are required for I_CVI and S_CVI, respectively, to confirm the validity of the content.

####  Face validity

 The face validity of the GTS was assessed from the perspective of the students using both quantitative and qualitative approaches.^22,23^ To qualitatively assess the Persian-GTS, 10 subjects were asked to evaluate and provide feedback on the difficulty, irrelevancy, and ambiguity in the items. Based on the feedback from the participants, the tool was revised to reduce its ambiguity and improve its clarity. Similarly, 15 subjects were asked to rate the importance of the items using a five-point Likert scale (one = not important to five = completely important). Next, the impact score of each item was calculated using the following formula: impact score = importance of item * frequency (%) of a similar item. The items with an impact score greater than 1.5 were considered to be suitable.

####  Construct validity

 The construct validity of the GTS was assessed using two methods: calculating the correlation coefficient between the scale and its subscales, and conducting confirmatory factor analysis (CFA).^24^ The maximum likelihood method was utilized for model estimation, and several fit indices were analyzed, including the chi-square index (χ^2^), chi-square ratio index (χ^2^/*df*), goodness of fit index (GFI), adjusted goodness of fit index (AGFI), comparative fit index (CFI), root mean square error of approximation (RMSEA), and root mean square residual (RMR). A non-significant χ^2^ value indicates a good fit, although this index tends to be significant in larger samples, which can make it less reliable for assessing fit. A ratio of χ^2^ to df below 3 suggests a very good fit. If the CFI, AGFI, and GFI values are greater than 0.90, and the RMSEA and RMR values are below 0.05, it indicates an excellent fit, while values below 0.08 suggest an acceptable fit. To estimate the sample size for CFA, general guidelines recommend having 2 to 10 samples for each item.^25,26^ In this study, 13 samples were recruited for each item.

###  Phase two: assessment of reliability 

####  Scale score reliability 

#####  Internal consistency reliability

 Cronbach’s alpha coefficient was computed to evaluate the internal consistency of the GTS. Alpha coefficient of at least 0.70 was considered acceptable.^27^

#####  Test–retest reliability

 To assess the reliability of the test-retest method, the intraclass correlation coefficient (ICC) was calculated for all items and domains. Participants who recently completed the GTS questionnaire were asked to complete it again two weeks later. The optimal time interval between the tests was determined by an expert panel, and a previous psychometric study.^28^ The time period between two tests should be long enough so that the subjects do not remember the previous answers. A 2-way mixed-effects model was used to calculate the ICC with a 95% confidential interval. An ICC of 0.75 and higher was considered indicative of appropriate stability.^29^ The smallest sample size needed was 50 to evaluate the intra-class correlation coefficient (ICC) value of 0.4, with a specified power of 90% and an alpha level of 0.05.^30^

###  Factor structure exploratory factor analysis (EFA)

**Figure 1:**
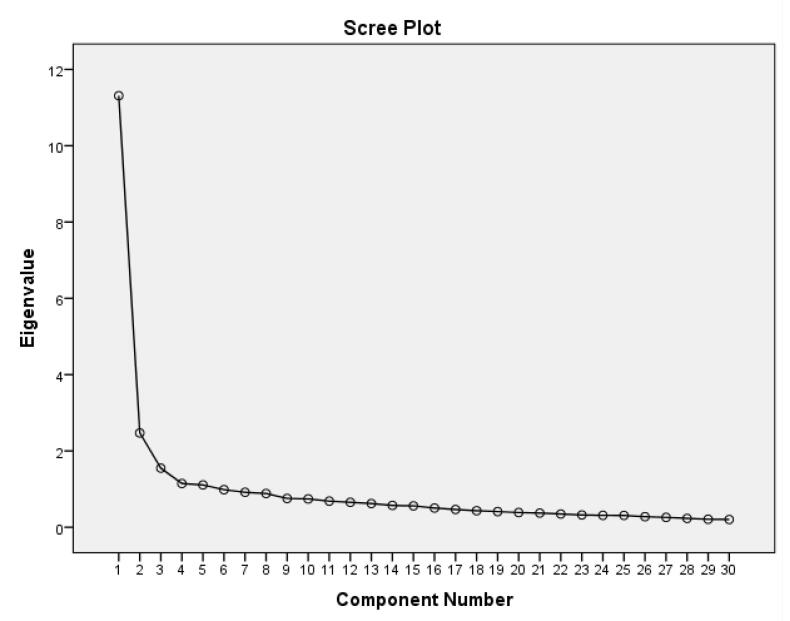
Scree plot of eigenvalues for factor retention of Persian Version of GTS

 An EFA utilizing principal component analysis and Oblimin with Kaiser normalization rotation method was employed to determine the optimal number of latent variables. To do so, EFA was conducted with a sample of 418 students, distinct from the samples used in the CFA (N = 418). A scree plot was employed to ascertain the number of factors (Figure 1).

###  Feasibility

####  Floor and ceiling effects 

 The presence of floor and ceiling effects was assessed by calculating the percentage of participants who received the minimum (32) and maximum possible scores (224) for each item on the tool. These effects were considered significant if they surpassed 15%.^31^

###  Statistical analysis 

 Mean and standard deviation was computed for quantitative variables with normal distributions. Data were analyzed using the IBM SPSS Statistics software package^32^ version 20 and LISREL software package, version 8.7.^33^

## Results

 A total of 418 individuals participated in the study, within which 204 cases (48.8%) were female. The mean age for female students was 23.31 (standard deviation [SD] = 1.92), and for male students was 23.72 (SD = 2.12). The content validity of the GTS was confirmed based on expert opinions. To improve the grammatical structure and phrasing of the items, researchers implemented minor modifications to the GTS items following expert recommendations. The CVR was determined to exceed 0.54 for all 32 items. The I_CVI was calculated to be equal to or greater than 0.8 for each item, while the S_CVI was found to be 0.95. According to feedback from 10 participants, there was no necessity to amend the items concerning their comprehensibility, clarity, and ease of completion. Given that the impact score for all items was above 1.5, no item was removed at this stage.

Table 1 shows, the mean and SD of the participants’ scores as well as the results of internal consistency and test-retest reliability for the subscales and total GTS scores. The average GTS score for the participants (n = 418) was 4.82 ± 1. The Cronbach’s alpha coefficient was determined to be 0.89 for the transphobia/genderism subscale, 0.83 for the gender-bashing subscale, and 0.91 for the overall GTS. Additionally, the ICC was calculated to be 0.99 for the transphobia/genderism subscale, 0.99 for the gender-bashing subscale, and 0.99 for the total GTS.

**Table 1:** Mean and standard deviation of the scales score and results of the internal consistency and test retest reliability

**Scale**	**No. of items**	**Mean**	SD*	**Cronbach's alpha coefficient**	**ICC (95%CI)**
Transphobia/genderism	23	4.51	1.05	0.89	0.99 (0.98-0.99)
Gender-bashing	7	5.84	1.14	0.83	0.99 (0.95-1)
GTS total	30	4.82	1.00	0.91	0.99 (0.94-0.99)

SD, Standard Deviation; GTS, genderism and transphobia scale; ICC, infraclass correlation coefficient.

 None of the participants reached either the minimum or maximum score on the GTS. Consequently, no ceiling or floor effect was observed for the Persian version of the GTS.

**Figure 2:**
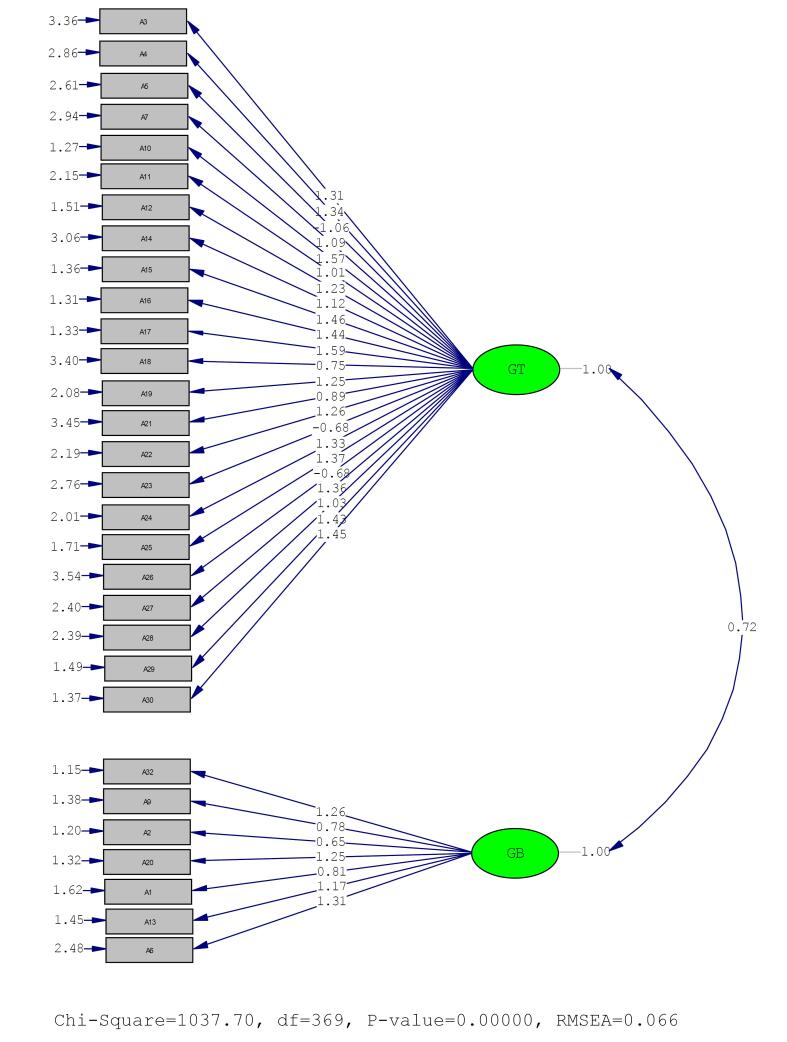
Standardized values of the two-factor confirmatory factor analysis of GTS (N = 418)

 The mean (SD) of the items, Cronbach’s alpha coefficients if each item is omitted, and the results of EFA are presented in Table 2. The results of EFA showed that the two factors of transphobia/genderism and gender-bashing collectively explained 47.05% of the total variance. We also found that the first and second factors explained 38.79% and 8.25% of the total variance, respectively. Since items number 8 and 31 had a factor loading of less than 0.2, they were removed from the Persian version of GTS. The Kaiser-Meyer-Olkin (KMO) measure for this analysis was 0.94, and Bartlett’s test of Sphericity yielded a value of 6777.2, which was statistically significant at *P* < 0.001. The fit indices of the two-factor GTS model indicated its optimal fit (Table 3). Figure 2 presents the path diagram of the model with standardized coefficients presented.

**Table 2:** Mean, standard deviation, Cronbach’s alpha if item deleted and factor loadings on explanatory principal components analysis of GTS scale items (n = 418)

**Scale**	**Item**	**Mean**	**SD**	**Cronbach's alpha if item deleted**		**Component**
					**1**	**2**
Transphobia/genderism	7	3.55	2.05	0.91	0.70	
	24	5.23	1.93	0.91	0.47	
	4	4.39	2.15	0.91	0.64	
	5	4.06	1.94	0.92	-0.64	
	17	4.52	1.97	0.91	0.79	
	30	4.68	1.86	0.91	0.73	
	15	4.78	1.87	0.91	0.74	
	27	4.08	2.07	0.91	0.67	
	25	4.48	1.89	0.91	0.71	
	19	3.82	1.91	0.91	0.71	
	16	5.27	1.84	0.91	0.64	
	3	4.53	2.24	0.91	0.63	
	29	5.00	1.87	0.91	0.64	
	26	4.29	2.00	0.92	-0.59	
	10	5.20	1.93	0.91	0.72	
	22	4.16	1.94	0.91	0.66	
	11	5.55	1.78	0.91	0.43	
	28	5.02	1.90	0.91	0.44	
	14	4.86	2.08	0.91	0.42	
	18	3.24	1.99	0.91	0.58	
	12	5.33	1.73	0.91	0.53	
	21	5.00	2.05	0.91	0.33	
	23	2.67	1.80	0.92	-0.31	
Gender-bashing	9	6.13	1.41	0.91		0.72
	2	6.38	1.29	0.91		0.74
	32	5.94	1.67	0.91		0.67
	20	5.68	1.72	0.91		0.70
	1	6.28	1.52	0.91		0.67
	13	5.68	1.68	0.91		0.61
	6	4.82	2.04	0.91		0.41

SD, Standard Deviation; GTS, genderism and transphobia scale.

**Table 3:** Model fit indices for GTS from confirmatory factor analyses

**Index of Fit**	**Indicator values**
Chi-square (χ^2^)	1037.70
χ^2^/df	2.81
Goodness of fit index (GFI)	0.86
Adjusted goodness of fit index (AGFI)	0.82
Comparative fit index (CFI)	0.98
Normed fit index (NFI)	0.96
Root mean square error of approximation (RMSEA)	0.06
Standardized root mean square residual (SRMR)	0.05
Relative fit index (RFI)	0.96
Incremental fit index (IFI)	0.98

## Discussion

 To the best of our knowledge, this is the first study examining the psychometric properties of the Persian GTS in an Iranian community. This research provides an opportunity to evaluate students’ attitudes toward transgender individuals and to facilitate comparisons of results across various contexts.

 As previously noted, transgender individuals encounter a multitude of challenges. In the United States, transgender populations experience harassment, discrimination, and violence. These individuals are subjected to both social/interpersonal stigma and structural stigma, which may collectively contribute to adverse physical and mental health outcomes.^7,34^ In addition to standard healthcare services, transgender individuals often require specialized treatments, including hormone therapy and surgical interventions. A study conducted in Turkey found that the individuals with female-to-male GD who underwent surgical procedures reported improvements in their social functioning.^35^

 Despite their special needs for care, they are often neglected in the health care system. Transgender individuals face numerous barriers when seeking healthcare services, including discrimination within the healthcare system, financial constraints, socioeconomic challenges, unsupported healthcare system frameworks, and a lack of specialized education among providers.^9,10^ Additionally, transphobia is not a rare issue among healthcare providers. Transgender individuals’ experiences of discrimination in healthcare settings include general discomfort, the use of harsh language or verbal/non-physical harassment, inappropriate name use, and outright denial or delay of healthcare services.^9^ These individuals often have stressful lives, which can predispose them to serious psychiatric disorders. They may also have experienced childhood abuse, which may lead to mood disorders, anxiety, self-harming behaviors, and personality disorders in later life.^36^ The content discussed above highlights the significance of conducting studies on the subject matter. Therefore, scales such as the GTS for assessing transphobia have been presented.

 The GTS questionnaire consists of two components: the first component measures transphobia and genderism, comprising 25 items, and the second component assesses sexism which consists of 7 items. This 32-item scale has been validated in three different studies, achieving a high level of internal consistency. The third study, unlike the two previous ones, demonstrated that a three-factor structure (transphobia, genderism, and gender bashing) was unsuitable for this scale, while a two-factor structure had greater internal consistency. The structure was then reduced to two factors: 25 items measure transphobia and genderism and 7 items measure gender bashing. Combining these two factors allows for the measurement of cognitive, behavioral, and affective dimensions of genderism, transphobia, and gender-based discrimination. Across these three studies, Cronbach’s alphas for the three subscales ranged between 0.79 and 0.95. In more detail, the developers reported Cronbach’s alpha coefficients of 0.83 for the sexism subscale, 0.94 for the transphobia subscale, and 0.79 for the gender panic subscale. These data show a high internal consistency value for the items.^37,38^

 GTS has been validated among different cultures. In the version of GTS validated in Hong Kong, three items of the original GTS (8, 26, and 16) were removed due to cultural and epidemiological reasons and their final questionnaire had 30 items. The authors grouped the items into 5 factors, and the results of the factor analysis showed Cronbach alpha coefficients ranging from 0.78 to 0.80. Remarkably, they could not calculate coefficients for two factors, due to a low number of items for those factors.^39^

 In Spain, Carrera-Fernández et al, also validated GTS in a shorter form in 2013. They removed items 8, 26, and 16 due to difficulties encountered in the Chinese adaptation. Additionally, items 18, 22, 28, and 31, which were ambiguous in the original adaptation, were removed. They eliminated the items that couldn’t accurately discriminate between adolescents with high and low scores in prejudice, thus they reduced number of the items to 12 for the short version of GTS. The short form of GTS showed good reliability, with an alpha coefficient of 0.80 for gender bashing and 0.83 for transphobia/genderism.^37^

 In another study, Tebbe et al evaluated the one-, two-, three-, and five-factor models posited or used in previous studies and concluded that the two-factor model had the strongest conceptual grounding. They also reformed GTS to a shorter form in which 10 items were eliminated due to the low level of factor loading and conceptual review of the items. They reported Cronbach’s alpha of 0.94 for genderism/transphobia subscale, 0.86 for gender bashing and 0.94 for the overall scale. They suggested that the refined form of GTS was more efficient than the original form producing equally reliable scores.^40^ In the current study, we validated the original 2-subscale form of GTS. Cronbach’s alpha coefficient for the internal consistency of the transphobia/genderism subscale was 0.89, and for the gender-bashing subscale was 0.83. The scale indicated a coefficient of 0.91 for the entire GTS, which shows a high level of internal consistency as reported by Hill and Willoughby.^16^

 We had to remove items number 8 (Children should be encouraged to explore their masculinity and femininity) and 31 (It’s all right to make fun of people who cross-dress), due to the factor loadings less than 0.3. These two items were from the genderism and transphobia subscale.^41^ As mentioned above, these items were eliminated in the Chinese version of GTS. It seems that the items are not align with Asian culture. Validating GTS among other populations of Asia may be helpful to confirm or reject this hypothesis. Removing these items from the scale in this study may be due to the lack of education about genderism in Iranian schools and families. Iranian parents are not so open with their children in the subjects associated with sex, which prevents children from receiving proper sexual education. In this context, family environment serves as a transition to society. As children explore and solidify their gender identity, they ready themselves for societal integration. Addressing these matters within the family provides children with a safe space to understand their gender. With parental support, they can mentally be prepared to face potential challenges. Moreover, resolving such issues within the family may facilitate easier acceptance of societal views, even if they initially seem negative. All these discussed emphasize the importance of validating GTS within Iranian population to assess the levels of transphobia in society. This validation is crucial for informing efforts aimed at educating families and society about transgender individuals, thereby making a more supportive environment. According to our findings, it is essential to incorporate transgender-specific health needs into medical school and residency curricula.

## Strengths and Limitations

 Our study had some limitations. The initial concern pertains to the carry-over effect associated with test-retest. To reduce the carry-over effect, a two-week interval was established between the test and retest, per the recommendations provided by psychologists and psychiatrists. The second limitation was related to the samples and the sampling method. The samples were recruited among medical sciences students using a convenience sampling approach. It is possible that these students, due to more information about transgender issues, have had more positive attitudes towards these individuals, compared to students from other universities. Consequently, this may affect the external validity of the findings. So, it is recommended to exercise caution when generalizing these results to other universities.

 Despite the limitations, this study serves as a baseline for future research. Further studies are recommended to include large samples from various universities and at the national level. Also, it is recommended that this tool is administered to both the general population as well as across different age groups.

## Conclusion

 In this study, we aimed to validate the GTS within the context of Iranian culture. We found it necessary to exclude items number 8 (“Children should be encouraged to explore their masculinity and femininity”) and 31 (“It’s acceptable to make fun of people who cross-dress”) due to analytical considerations. Excluding these items may highlight a cultural gap in information within families and society regarding sexual education, including education about sexual identity.

 Finally, the findings of the present study indicate that the Persian version of GTS demonstrates both validity and reliability in evaluating students’ attitudes toward transgender individuals. The instrument showed adequate levels of face and content validity, as well as strong internal consistency and stability, suggesting that it may effectively measure attitudes about GT, and provides highly reliable scores at both the item and total score levels within such samples. Therefore, the Persian GTS now can be used in the studies on planning for health issues associated to transgender individuals.

## Competing Interests

 The authors have no competing interests to declare.

## Ethical Approval

 This study is a non-interventional ethical study that was conducted under the supervision of the ethics committee of Tabriz University of Medical Sciences after obtaining an ethical code (IR.TBZMED.REC.1402.042). Prior individuals responded to the questionnaire, they were given information about the research process. They were also informed of their right to exit the research at any time if they lost interest in participating. Provided questionnaires were anonymous and the information of the participants has been kept confidential. The statistical analysis of the research was impeccable, coordinated with the data extracted from questionnaires, and without any impact of personal judgment.
